# SKL1 Is Essential for Chloroplast Development in Arabidopsis

**DOI:** 10.3389/fpls.2018.00179

**Published:** 2018-02-20

**Authors:** Huimin Xu, Liwen Zhang, Ruili Li, Xinwei Wang, Shuai Liu, Xiaomin Liu, Yanping Jing, Jianwei Xiao

**Affiliations:** ^1^Beijing Advanced Innovation Center for Tree Breeding by Molecular Design, Beijing Forestry University, Beijing, China; ^2^College of Biological Sciences and Biotechnology, Beijing Forestry University, Beijing, China; ^3^College of Life Sciences, Peking University, Beijing, China

**Keywords:** SKL1, chloroplast, development, Arabidopsis, gene expression

## Abstract

The Arabidopsis *shikimate kinase-like 1* (*skl1-8*) mutant is characterized by a pigment-defective phenotype. Although the related phenotypical defect mainly has been attributed to the blocking of chloroplast development, the molecular functions of SKL1 remain largely unknown. In this study, we combined multiple approaches to investigate the potential functions of SKL1. Results showed that the *skl1-8* mutant exhibited an albino phenotype and had dramatically reduced chlorophyll content as a consequence of a single nuclear recessive gene mutation. Chemical complementation analysis indicated that SKL1 does not function as SK enzyme in the shikimate pathway. In addition, by chlorophyll fluorescence parameters and immunoblot analysis, the levels of photosynthetic proteins are substantially reduced. Moreover, by transcriptome analysis, specific groups of nuclear genes involved in photosynthesis, such as light-harvesting complex, pigment metabolism, carbon metabolism, and chloroplast gene expression, were down-regulated, whereas several defense and oxidative stress responsive genes were up-regulated in the *skl1-8* mutant compared with the wide type. Furthermore, we found the expression of genes related to auxin transport and response was repressed in the *skl1-8* mutant, probable suggesting that SKL1 is involved in auxin-related pathways during chloroplast development. Together, these results provide a useful reference for characterization of SKL1 function during chloroplast biogenesis and development.

## Introduction

Chloroplasts are organelles, specialized subunits, in plant and algal cells. The most important role of chloroplast is to conduct photosynthesis and related productions are critical for plant growth (Abdallah et al., 2000; Peltier et al., 2006). As the main site of photosynthesis, chloroplasts rely on the energy captured by photosynthetic pigment chlorophyll to fix carbon dioxide (Waters and Langdale, 2009), and numerous important biosynthetic pathways are carried out in the chloroplast, including tetrapyrroles, terpenoids, fatty acid biosynthesis, amino acids biosynthesis, hormones, and the immune response in plants (Givan and Stumpf, 1971; Kusumi et al., 2004; Seay et al., 2009; Hou et al., 2016).

Chloroplasts, which originated from cyanobacterial ancestors, are semiautonomous organelles with their own genome in higher plants (Martin et al., 2002; Hanaoka et al., 2003). The most photosynthetic proteins are nuclear encoded, synthesized in cytoplasm as precursor proteins and being imported into the chloroplast (Inaba and Schnell, 2008), it is has been confirmed that the disruption on nuclear-encoded and chloroplast-localized proteins can specifically disrupts the chloroplast biogenesis by alternative biological functions, such as chloroplast gene expression, protein import, complexes assembly, lipid biosynthesis and so on (Gutiérrez-Nava et al., 2004; van Wijk, 2015; Zheng et al., 2016; Wang et al., 2017; Zhang Z. et al., 2017). Therefore, studies on such mutants would be further elucidating the regulatory mechanisms of chloroplast biogenesis and development. By investigating the SeedGenes database, a total of 119 chloroplast proteins encoded by nuclear genes were found to participate in embryo development in Arabidopsis (Tzafrir et al., 2004). Then reported proteins can be roughly classified into three groups based on their potential roles: (i) proteins required for the localization and modification of chloroplast proteins; (ii) enzymes are necessary for the biosynthesis of vitamins, amino acids, fatty acids and nucleotides; and (iii) regulated factors participated in the plastid genes expression (Bryant et al., 2011).

Among the nuclear-encoded factors that are involved in chloroplast and embryo development, the *shikimate kinase-like 1-8* (*skl1-8*) mutant was first described by characterization of an albino phenotype of a T-DNA-inserted allele of *AtSKL1*, and it was reported that SKL1 is essential for chloroplast development (Fucile et al., 2008). The *SKL1* gene evolved from Shikimate kinase (SK) gene duplicates more than 400 million years ago and can be found in all major extant angiosperm lineages (Fucile et al., 2008; Maeda and Dudareva, 2012). The SK proteins are involved in the intermediate step of the shikimate pathway, which is closely linked with produced processes of the aromatic amino acids (Herrmann, 1995). Considerable studies have been carried on the shikimate pathway because the three aromatic amino acids cannot be produced by some livestock and humans (Tzin and Galili, 2010). It has been estimated that 20% of the carbon fixed by plants can be directed toward the shikimate pathway (Herrmann, 1995), and this pathway is usually confined in chloroplast (Mustafa and Verpoorte, 2005). As the ancient homologous protein to SK, the recombinant SKL1 is purified in the same manner and showed a similar stable level as the active AtSK1, but SKL1 does not possess SK enzyme activity *in vitro* (Fucile et al., 2008).

SKL1 has been implicated in plastid biogenesis in Arabidopsis, however, a number of questions still remain unresolved, such as the exact function in chloroplast development and plastid biogenesis. In this study, we address these questions by combining a phenotypic and transcriptome analysis to further our understanding in the mechanism of SKL1 in the chloroplast development.

## Materials and Methods

### Plant Materials and Growth Conditions

Surface-sterilized wild type and *skl1-8* mutant seeds were sown on 1/2 Murashige and Skoog (MS) agar Petri plates, and placed in the dark at 4°C for 48 h to imbibe. After germination, the plants moved to a growth chamber maintained at 22°C under long day conditions (16 h light, 8 h dark). For chemical complementation analysis, we resuspended shikimate 3-phosphate (Sigma, St. Louis, MO, United States) and chorismate (Sigma, St. Louis, MO, United States) in water at a concentration of 50 mM and next added it to the MS medium at a final working concentration of 50 μM.

### Complementation of the *skl1-8* Mutant

To complement the s*kl1-8* mutant, we amplified a full-length cDNA fragment encoding SKL1 by reverse transcription polymerase chain reaction (RT-PCR) using the specific primers from wild type. The primers sequences are list in the **Supplementary Table S1**. The PCR product was digested and subcloned into the pSN1301 vector by controlling of the cauliflower mosaic virus 35S promoter. Through the floral dip method, the construct was introduced into heterozygous *skl1-8* plant (Clough and Bent, 1998). The transgenic Arabidopsis was selected using 50 mg L^-1^ hygromycin medium and successful complementation was confirmed by PCR analysis.

### Measurement of Chlorophyll

To measure the chlorophyll content, the whole Arabidopsis leaves were collected from three individual WT and *skl1-8* seedlings, respectively. We extracted the chlorophyll in 80% acetone and brief centrifugation (12000 *g*) at 4°C and quantified using a UV2800 spectrophotometer (Unico, Dayton, NJ, United States). We calculated the chlorophyll content from the absorbance following the method of Arnon (1949).

### Total Protein Preparations and Immunoblot Analysis

In immunoblot analysis, total proteins were extracted from Arabidopsis leaves and separated by SDS–polyacrylamide gel electrophoresis (PAGE), and then were transferred to nitrocellulose membranes. The membranes were incubated with specific primary antibodies, and signals from secondary conjugated antibodies were visualized using the enhanced chemiluminescence method. We scanned X-ray films using an AlphaImager 2200 documentation and analysis system (Alpha Innotech Corporation, San Leandro, CA, United States). The antibodies referred in this analysis according to Zhang J. et al. (2017).

### Chlorophyll Fluorescence Measurements

We measured the chlorophyll fluorescence by using a CF Imager (Technologica, Essex, United Kingdom) and operated it as described by Xiao et al. (2012). In briefly, the leaves were first dark adapted for 10 min before each measurement and the minimum fluorescence yield (*F*_o_) was measured with a measuring light intensity of 0.8 mmol m^-2^ s^-1^. For measuring the maximum fluorescence yield (*F*_m_), we applied a saturating pulse of white light to the plants for 1 s, and the light intensity is 3000 mmol m^-2^ s^-1^. We calculated the maximal photochemical efficiency of PSII by the ratio of *F*_v_ (*F*_m_ – *F*_o_) to *F*_m_. In image analysis, the corresponding data measured in the plants were normalized to a false-color scale with assigned extreme highest value with 0.8 (red) and lowest value with 0.1 (blue), respectively.

### RNA Isolation, cDNA Library Preparation, and Transcriptome Sequencing

The method used in the transcripome analysis according to Xue et al. (2017). In general, sequencing libraries were generated using the NEBNext^®^ Ultra^TM^ RNA Library Prep Kit for an Illumina^®^ device (NEB, Ipswich, MA, United States). Three cDNA libraries each for *skl1-8* mutant and wild type were constructed and sequencing by using the Illumina HiSeq 2500 platform. We pooled the clean reads of the three libraries for *de novo* assembly of the global transcriptome using Trinity (Grabherr et al., 2011). We cross-checked all of the assembled unigenes against the NR database using the Basic Logic Alignment Search Tool algorithm with an *E*-value cut-off of 10^-5^ (Korf et al., 2003).

Differentially expressed genes (DEGs) were identified in *skl1-8* and wild type according to the |log_2_ (fold-change)|≥ 1 with the *p*-value < 0.05. The expression level for each unigene was calculated and normalized using the RPKM (reads per kb per million reads; Mortazavi et al., 2008). In addition, Gene Ontology Enrichment Analysis Software Toolkit were used to multi-GO enrichment analyses of DEGs (Zheng and Wang, 2008).

### Quantitative Real-Time PCR

We synthesized first-strand cDNA using Transcript One-Step gDNA Removal and cDNA Synthesis SuperMix (TianGen Biotech, Beijing, China) following the instructions of manufacturer. Three samples were obtained from the wild-type and *skl1-8* mutant leaves, respectively. We conducted real-time PCR reactions containing 2 μL of first-strand cDNA in a total volume of 20 μL. Relative quantities of expression levels of the genes were calculated using the 2^-ΔΔCt^ method (Livak and Schmittgen, 2001) and the *Actin* gene used as internal control. We using a LineGene 9600 detection system to measure the quantity of amplified DNA by the fluorescence produced in the end of PCR reaction (BIOER, Hangzhou, China). The measurement for each sample was repeated in three times and the primer pairs used in this analysis listed in **Supplementary Table S1**.

## Results

### Phenotype of *skl1-8* Mutant

**FIGURE 1:**
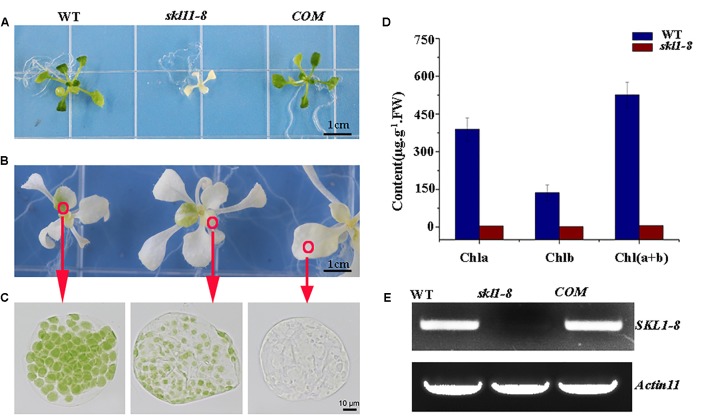
Identification of the *skl1-8* mutant. **(A)** Identification and complementation of the *skl1-8* mutant. The cDNA of the wild-type *SKL1* with the flag sequence was cloned into a binary plant transformation vector and used for complementation of the *skl1-8* mutant (*COM*). WT, wild type. 14-day-old plants, including WT, *skl1-8*, and *COM*, were grown on sucrose-supplemented medium. Scale bar: 3 mm. **(B)** Different yellow-green phenotype in *skl1-8* mutant. **(C)** Chloroplast phenotypes of mesophyll cells in *skl1-8* mutant with different yellow–green phenotype. **(D)** Reverse transcription (RT)-PCR analysis. RT-PCR was performed using specific primers for *SKL1* or *ACTIN11*. **(E)** Mean values of chlorophyll contents (Chla and Chlb) in WT and *skl1-8* mutant seedlings. Leaf tissue was sampled from 14-day-old plants and data are mean values of three measurements ± SD.

The *skl1-8* mutant was first characterized by Fucile et al. (2008), and we got this mutant from Arabidopsis Biological Resource Center. The *skl1-8* mutant exhibited an albino phenotype and was obviously smaller in size relative to the wild type (**Figure 1A**). To confirm whether the phenotype of the *skl1-8* mutant was due to the disruption in *SKL1*, we transformed the full-length coding region of *SKL1* gene into the *skl1-8* mutant. After 14 days of growth, we observed *COM* transgenic plant showing similar leave color and plant size to the wild type (**Figure 1A**). When cultivated in sucrose-supplemented medium for 4 weeks, some white parts of the leaf in *skl1-8* mutant developed to different shades of yellow and green patches (**Figure 1B**). In addition, we also observed chloroplast phenotypes of different sections of the mesophyll cells with varied green parts in the *skl1-8* mutant (**Figure 1C**), showing that the chloroplast was more seriously affected in the white part than in the green part. Furthermore, we measured the chlorophyll content in 4-week-old *skl1-8* mutant and exhibited dramatically reduced levels compared with wild type (**Figure 1D**). Eventually, *COM* plants accumulated substantial quantities of chlorophyll, almost reaching the wild-type levels. RT-PCR analysis showed that the transcript of the *SKL1* gene was barely detected in s*kl1-8* mutant, whereas obvious signals were obtained from *COM* and wild-type plants (**Figure 1E**). These results indicating that *skl1-8* mutant is null and *SKL1* gene was responsible for the phenotype of the *skl1-8*.

### Homozygous *skl1-8* Mutant Cannot Be Rescued by Intermediates in Shikimate Pathway

**FIGURE 2:**
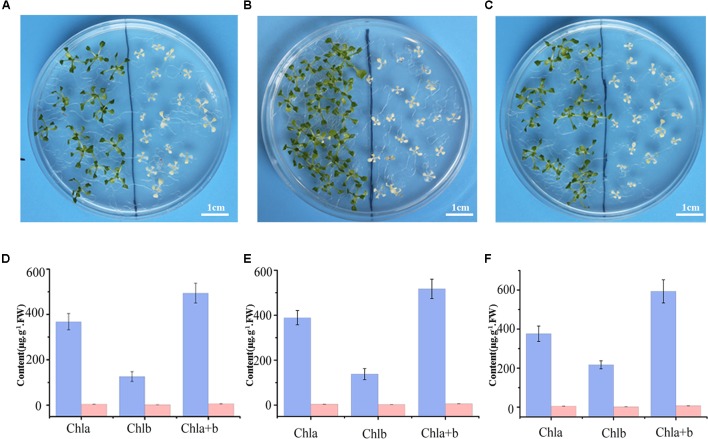
Complement analysis of compound intermediates in the shikimate pathway. Phenotype of wild-type (left), and *skl1-8* seedlings (right) grown in **(A)** the regular MS medium, **(B)** the presence of chorismate, and **(C)** shikimate 3-phosphate. The chlorophyll contents in Arabidopsis seedlings were showed in **(D–F)**, respectively, and the data are mean values of three measurements ± SD.

Previous research suggested SKL1 to be an ancient homologous protein with SK proteins (Fucile et al., 2008), leading us to investigate whether SKL1 plays a similar role as SK in the shikimate pathway. We analyzed the phenotype of young seedlings of *skl1-8* mutant by growth in a complement of compound intermediates in the shikimate pathway. We first germinated *skl1-8* and wild-type plants on regular MS medium and then transferred them to MS medium supplemented with either chorismate or shikimate 3-phosphate. The materials transferred to regular MS medium were used as control. After cultivated for 2 weeks and compared with the control, the albino phenotype of *skl1-8* mutant showed no visible changes whatever grown on chorismate or shikimate 3-phosphate containing medium (**Figures 2A–C**).

Meanwhile, the pigment profiles of wild-type plant and *skl1-8* mutant are similar to the corresponding parts grown on regular MS medium (**Figures 2D–F**). This result indicates that the phenotype of *skl1-8* cannot be recovered when grows in the presence of chorismate or shikimate 3-phosphate. Thus, the combination of phenotypic and chlorophyll analysis excludes the fact that SKL1 function as SK proteins in the shikimate pathway.

### Immunoblot Analysis of Chloroplast Proteins in *skl1-8* Mutant

**FIGURE 3:**
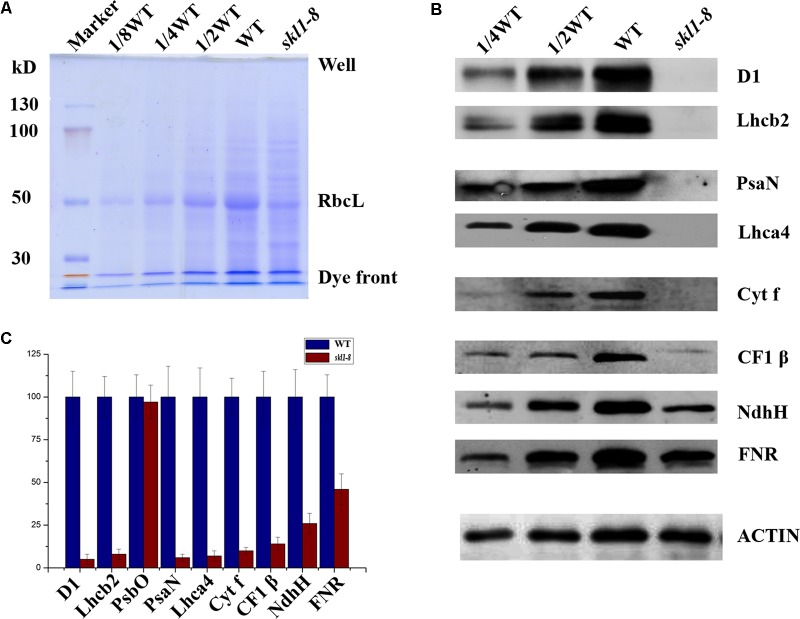
Accumulation of chloroplast proteins. **(A)** Coomassie brilliant blue staining of total proteins. Total protein resolved by SDS/PAGE from WT and *skl1-8* and the major band of RbcL was indicated. **(B)** Western blot analysis of D1, Lhcb2, PsaN, Lhca4, Cyt f, CF1β, NdhH, FNR, and ACTIN in the total protein extract from wild-type and *skl1-8* plants. These experiments were repeated three times independently, and similar results were obtained each time. Results from a representative experiment are shown. **(C)** The average signal intensities for each protein were quantified by the ImageJ software for three independent times, expressed level relative to the WT^∗^ (=100), and are indicated on the left.

Our previous studies have shown that the reduced content of chlorophyll is always coupled with defective accumulation of photosynthetic protein complexes (Zhang J. et al., 2017). To test this possibility, we analyzed the protein profile to further investigate the stable level of photosynthetic proteins in *skl1-8* mutant. By coomassie brilliant blue staining, we observed a significant reduction in the content of the large subunit of ribulose bisphosphate carboxylase (RbcL) in *skl1-8* mutant (**Figure 3A**). We also performed western blot experiment to analyze the proteins involved in photosynthesis, including core subunit of photosystem II, D1; N subunit of photosystem I, PsaN; the light-harvesting chlorophyll binding protein2, Lhcb2; β subunit of the ATP synthase, CF1 β; H subunit of Ndh complex, NdhH; and major subunit of Cyt b6/f complex, Cyt f. D1, Lhcb2, PsaN, Lhca4, and Cyt f were all barely detectable in *skl1-8* mutant, and CF1 β and NdhH were dramatically reduced (**Figures 3B,C**). A component of photosynthetic electron transport chain, FNR, was reduced about 50% of the wild type (**Figures 3B,C**). The significant reduction in representative subunits of photosynthetic complexes was detected by western blot analysis revealed that the thylakoid membrane was very likely disrupted and resulted in albino phenotype in *skl1-8* mutant.

### Chlorophyll Fluorescence Analysis

**FIGURE 4:**
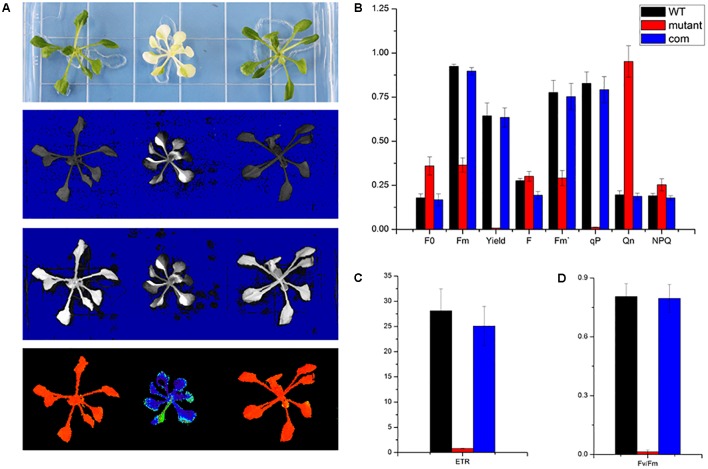
Photosynthetic fluorescence parameters of WT, *skl1-8* mutant, and *COM.* **(A)** Fluorescence images of WT, *skl1* mutant, and *COM*. **(B)** The values of minimum fluorescence (*F*_o_), maximum fluorescence (*F*_m_), and *F*_o_/*F*_m_; effective quantum yields of PS (Yield); photochemical quenching (qP); and quantum yield of regulated energy dissipation (NPQ). **(C)** Relative electron transport rates (ETRs). **(D)** *F*_v_/*F*_m_ ratios. The above experiments were repeated three times independently.

As a non-invasive signature of photosynthesis, chlorophyll fluorescence was usually used to measure the functional status of the photosynthetic apparatus (Mohammed et al., 1995). Therefore, the fluorescence parameters *F*_o_, *F*_m_, *F*_v_/*F*_m_, quantum yield of photosystem II (Yield), photochemical quenching (qP), and electron transport rate (ETR) were recorded by a pulse amplitude modulation fluorometer (PAM-2100; Heinz Walz GmbH, Effeltrich, Germany). We measured the chlorophyll fluorescence in wild type, *skl1-8*, and *COM* grown on MS medium for 4 weeks and studied their photosynthetic photochemical image (**Figure 4A**). Compared with that of wild type, the Yield, *F*_m_, and F′_m_ in *skl1-8* mutant decreased by 99, 60, and 63%, respectively (**Figure 4B**). Photochemical quenching (qP) measures the fraction of active PSII reaction centers. In *skl1-8* mutant, qP values were close to zero, and qP increased pronouncedly in *COM* plants nearly about to wild-type level (**Figure 4B**). In addition, the ETR of *skl1-8* mutant also decreased dramatically compared with the wild type (**Figure 4C**). The *F*_v_/*F*_m_ ratio reflects the maximum capacity of absorbed quanta in PSII reaction centers, and in *skl1-8* mutant, compared with the wild type, the *F*_v_/*F*_m_ ratio decreased by 98%, but in *COM* plants almost recovered to normal level (**Figure 4D**), suggesting that the photosynthetic efficiency was severely affected in *skl1*-8 mutant.

### Transcriptome Analysis of *skl1-8* Mutant Revealed Concerted Changes in Gene Expression

From the previously described chemical and physiological features (Zhang J. et al., 2017), we proposed that the expression pattern of genes responsible for photosynthesis and chloroplast development may have been altered in *skl1-8* mutant. To test this hypothesis, we subjected cDNA libraries from wild-type Arabidopsis and *skl1-8* mutant to Illumina sequencing. After removing invalid reads and data cleaning, we obtained 53101230, 70398388, 51095958 and 52035280, 51115882, 45617702 clean reads from three constructed libraries of *skl1-8* and wild type, respectively. The proportion of nucleotides with quality values larger than 20 in reads (the Q20 percentages) were 97.34 and 97.45, and the guanine and cytosine (GC) percentages were 46.07 and 45.73% (**Table 1**).

**Table 1:** Summary for transcriptome data of wild-type *Arabidopsis thaliana* and *skl1*-8 mutant.

Sample name	Raw reads	Clean reads	clean bases	Error rate (%)	Q20 (%)	Q30 (%)	GC content (%)
WT_1	53101230	53101230	7.97G	0.01	97.81	94.2	45.86
WT_2	70398388	70398388	10.56G	0.01	97.7	93.96	46.06
WT_3	51138598	51095958	7.66G	0.02	96.52	90.99	46.31
*skl1*_1	52035280	52035280	7.81G	0.01	97.86	94.32	45.45
*skl1*_2	51115882	51115882	7.67G	0.01	97.81	94.21	45.43
*skl1*_3	45655910	45617702	6.84G	0.02	96.69	91.3	46.33

**FIGURE 5:**
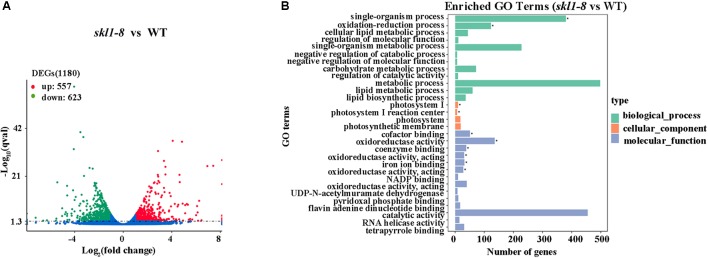
Differential expression analysis. **(A)** Diagram showing the number of significantly differentially expressed genes (*P*-value < 0.05, FC > = 1) in *skl1-8* and WT. **(B)** differentially expressed genes GO enriched pathway.

We identified a total of 1180 significant DEGs, consisting of 557 upregulated genes and 623 downregulated genes according to the RPKM calculation (**Figure 5A** and **Supplementary Table S2**). We classified all the functional DEGs and unigenes based on the gene ontology (GO) database through Blast2GO suite. We classified the DEGs into three different groups: biological process, molecular function and cellular component (**Figure 5B**). Importantly, we found that the oxidation-reduction process (123 genes) and the single-organism metabolic process (228 genes) involved in the biological process were significantly different, as well as the oxidoreductase activity (136 genes) involved in the molecular processes (**Supplementary Table S2**).

### Photosynthesis-Related Genes Expression Affected in *skl1-8* Mutant

In higher land plants, photosynthesis is a complex process involving a highly coordinated gene expression between nuclear and chloroplast genome. Identification and characterization of the DEGs within chloroplast-related mutants would be helpful in elucidating the mechanism of photosynthetic development and sustained autotrophic growth.

**FIGURE 6:**
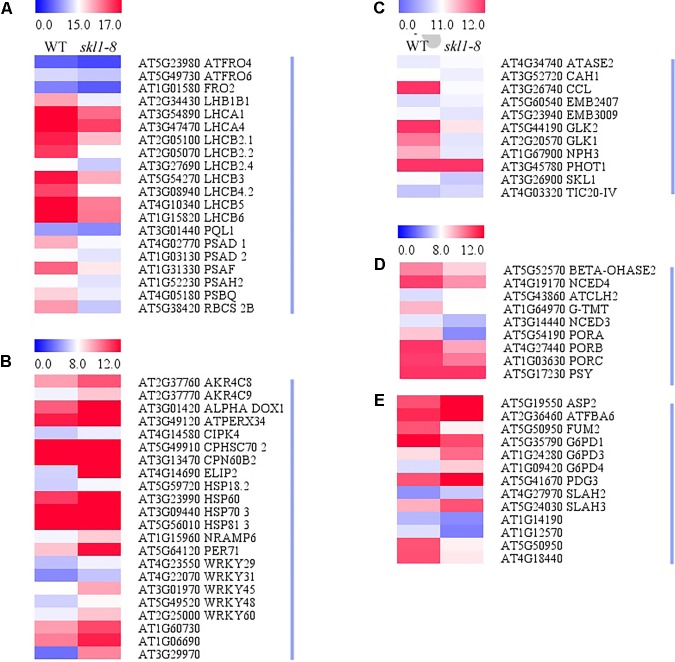
Heat map of DEGs in WT and *skl1-8*. **(A)** The genes related to photosynthesis and light-harvesting complex. **(B)** The response of genes to stress. **(C)** Genes related to photosynthetic regulation. **(D)** Pigment metabolism. **(E)** Carbon metabolism. The bar represents the scale of the expression levels for each gene (log_2_ FPKM) in WT and *skl1-8* as indicated by red/blue rectangles. Red indicates upregulation [log_2_ (fold change) ≥ 1.0, *p*-value < 0.05], green indicates downregulation [log_2_ (fold change) ≤ –1.0, *p*-value < 0.05] as compared with the wild type. The details of each gene are presented in **Supplementary Table S2**.

Our study showed that four protein subunits of LHCII proteins surrounding the PSII core were all significantly suppressed in *skl1-8* mutant, including *LHCB4-2*, *LHCB2-4*, *LHCB2-2*, and *LHCB2-1*, as well as *RBCS-2B* (gene encoded small subunit of RUBISCO; **Figure 6A**). By contrast, several of the genes involved in oxidative and other stress, such as some heat-shock proteins (i.e., *HSP70*) and *CPN60B2*, were highly activated in *skl1-8* mutant (**Figure 6B**). In addition, a high transcript level of *ELIP2* (*AT4G14690*), and the WRKY family transcription factors was observed in *skl1-8* mutant (**Figure 6B**). Furthermore, it is worth noting that a large number of key genes related to photosynthetic regulation in *skl1-8* mutant, such as *GLK1*, *GLK2*, *CCL*, *PHOT1*, and *NPH3* were downregulated (**Figure 6C** and **Supplementary Table S2**), conversely, *TIC20-IV* and *CAH1*, are involved in the nuclear-encoded proteins import into chloroplast, are activated in *skl1-8* mutant (**Figure 6C** and **Supplementary Table S2**). As expected, the level of *SKL1* (*AT3G26900*) transcripts was greatly reduced about 3.53-fold in *skl1-8* mutant compared with wild type (**Figure 6C** and **Supplementary Table S2**).

In the present study, genes related to pigment biosynthesis, such as light-dependent protochlorophyllide reductase (*PORA, PORB*, and *PORC*), *ATCHL2*, Tocopherol *O*-methyltransferase (*G-TMT*) and carotenoid oxygenase (*NCED4*) were all suppressed (**Figures 6D,E** and **Supplementary Table S2**), which was consistent with the albino leaf phenotype of *skl1-8*. Altogether, the result of the transcriptome analysis indicated that the reduced level of photosynthesis-related genes were responsible for defective chloroplast in *skl1-8* mutant, and meanwhile to modulate the expression of corresponding genes to deal with insufficient energy supply.

### Expression Patterns of Genes Response to Biosynthesis of Secondary Metabolites and Plant Hormones

**FIGURE 7:**
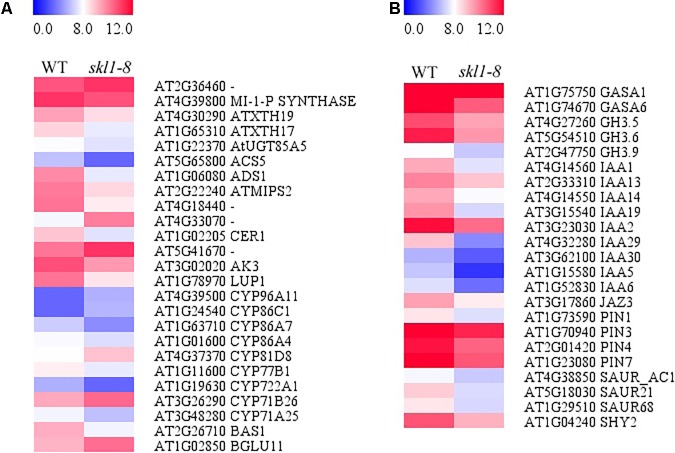
Analysis of DEGs in **(A)** biosynthesis of secondary metabolites and **(B)** plant hormone signal transduction. Red indicates upregulation [log_2_ (fold change) ≥ 1.0, *p*-value < 0.05], and green indicates downregulation [log_2_ (fold change) ≤ –1.0, *p*-value < 0.05] as compared with the wild type. The details of each gene are presented in **Supplementary Table S2**.

We analyzed the DEGs in biosynthesis of secondary metabolites (**Figure 7A**), including Fatty acid desaturase (*ADS1*), Fatty acid hydroxylase (*CER1*) and Pyridoxal phosphate-dependent transferase (*ACS5*) appeared to be repressed in *skl1-8* mutant. The expression of *ACS5* in *skl1-8* mutant had an obvious 4.8-fold change compared with wild type (**Figure 7A** and **Supplementary Table S2**). Some genes associated with cell wall biosynthesis response to secondary metabolites were also downregulated, such as xyloglucan endotransglucosylase (*ATXTH19*, *ATXTH17*), UDP-glucosyltransferase (*AtUGT85A5*), Terpenoid cyclases (*LUP1*). Conversely, components of the secondary metabolites signaling pathway, glycoside hydrolase (*BGLU11*) and thiamine pyrophosphate enzyme, were obviously upregulated in *skl1-8* mutant (**Figure 7A** and **Supplementary Table S2**).

By transcriptome analysis, we found that the hormone signal transduction pathways were altered in *skl1*-8 mutant (**Figure 7B** and **Supplementary Table S2**). Strikingly, most auxin-related genes, such as *IAA1*, *IAA2*, *IAA19*, and *IAA29*, showed an especially lower level of transcripts in *skl1-8* mutant (**Supplementary Table S2**), and similarly, the auxin efflux carrier, including *PIN3* and *PIN4*, were downregulated (**Figure 7B** and **Supplementary Table S2**). The altered expression patterns of genes related secondary metabolites and plant hormones provided a clue for elucidation for the mechanism of SKL1 involved in chloroplast development.

### Gene Expression Profiles Confirmed by qRT-PCR

**FIGURE 8:**
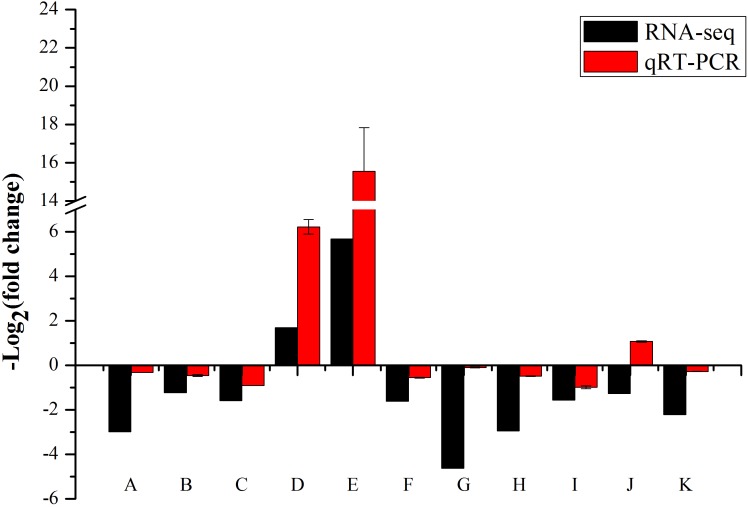
Validation of RNA-seq results by RT-qPCR. The 19 points (A–K) from left to right on the *x*-axis represent genes encoding *LHCB2.4* (*AT3G27690*), *LHCB2.1* (*AT2G05100*), *LHCB2.2* (*AT2G05070*), *HSP70-3* (*AT3G09440*), *ELIP2* (*AT4G14690*), *NCED4* (*AT4G19170*), *IAA29* (AT4G32280), *IAA19* (*AT3G15540*), *PIN3* (*AT1G70940*), *PIN4* (*AT2G01420*), *IAA1* (*AT4G14560*). The measurement for each sample was repeated in three times.

We performed the real-time RT-PCR analysis to confirmed the transcriptome result about *SKL1* mutation on photosynthetic gene expression. We first analyzed the transcript levels of nucleus-encoded photosynthetic genes, containing *LHCB2.4*, *LHCB2.1*, and *LHCB2.2*, which were light-harvesting complex II proteins and were downregulated in the *skl1-8* mutant compared with the wild type (**Figure 8**). Furthermore, *HSP70-3* and *ELIP2*, were upregulated, and the other genes, such as *NCED4*, *IAA29*, *IAA19*, *PIN3*, *PIN4*, and *IAA1* were all repressed in the *skl1-8* mutant (**Figure 8**). In total, we selected 11 genes as candidates for qRT-PCR analysis, in agreement with the transcriptome analysis.

## Discussion

We investigated the role of the SKL1, an ortholog of SK protein located in Arabidopsis chloroplast, which was shown to be responsible for early chloroplast development and plant growth (Fucile et al., 2008). In this study, we found the phenotype and chlorophyll content were dramatically affected in *skl1-8* mutant, a feature that highlighted the critical role of SKL1 during early plant development. Our current result demonstrated that SKL1 is required for the accumulation of four complexes related the photosynthesis. By inactivation of *SKL1* in Arabidopsis, we confirmed that SKL1 leads to a considerable decrease in photosynthetic performance.

The shikimate pathway is widely found in plants and bacteria and it is crucial for the biological synthesis of the aromatic amino acids required for the synthesis of other compounds in secondary metabolic pathways (Herrmann, 1995). In the shikimate pathway, SK is the key enzyme which can phosphorylate shikimate into shikimate 3-phosphate (Herrmann, 1995). As a homolog of SK proteins, SKL1, probably possesses the similar enzyme activity and affects the compounds’ biosynthesis in chloroplast. By complementation analysis, our result consisted with previous findings and confirmed that SKL1 does not perform a SK enzyme activity in Arabidopsis. It has been reported that SKL1 possesses a phosphoglycerate mutase-like (PGML) domain which conserved in phosphoglycerate mutase (PGAM) amino sequence (Fucile et al., 2008). In the glycolysis pathway, PGAM is a critical enzyme, and catalyses the interconversion of the phosphate group from 3 to 2-PGA. Andriotis et al. (2010) reported that total six genes, including *At1g22170*, *At3g08590*, *At1g09780*, *At1g78050*, *At3g30840*, and *At4g09520*, encode putative PGAM proteins, and proteins At1g22170 and At3g08590 were confirmed to be localized in chloroplast (Andriotis et al., 2010). Followed studies indicated that none of the single mutant of *At1g09780*, *At1g22170*, or *At3g08590* showed any visible morphological phenotypes compared with wild-type plants (Andriotis et al., 2010; Zhao and Assmann, 2011). Although we cannot exclude the possibility that SKL1 functions as PGAM in the chloroplast by present data, and according to above researches, it is hardly to attributed the serious phenotype in *skl1-8* mutant as a result of dysfunctional PGAM enzyme activity.

The reduced transcript level in photosynthetic genes always closely linked with albino phenotype in Arabidopsis (Zhang J. et al., 2017). Thus, we performed transcriptome analysis and identified a total of 1180 DEGs in *skl1-8* mutant, including 557 upregulated genes and 623 downregulated genes. Consistent with our original prediction, *skl1-8* mutant showed a differential transcript level of genes related to photosynthetic performance, such as gene expression (*GLK1*, *GLK2*, and *ATASE2*), protein transport (*TIC20-IV* and *CAH1*), chlorophyll biosynthesis (*PORA*, *PORB*, and *PORC*), and pigment biosynthesis (*PSY* and *NCED4*). In agreement with the notion that the defective chloroplast affects the transcription of nuclear-encoded chloroplast proteins via retrograde signaling (Chi et al., 2013), and in our results, dozens of nuclear genes that are relate to chloroplast development and photosynthesis are suppressed in *skl1-8* mutant. Thus, disrupted expression of related genes has potential responsiblity for the decreasing photosynthetic capability and abnormal growth of *skl-8* mutant.

Of most importance, by interacted with assistant proteins, chlorophyll pigments can be assembled into the light harvesting complexes, which capture light energy and transfer it to centers of PSII and PSI (Liu et al., 2004). On the basis of the transcriptome database, we found that LHC-encoding genes (*LHCB4.2*, *LHCB2.4*, *LHCB2.1*, and *LHCB2.2*) and *RBCS-2B* were classified into DEGs, showing reduced expression levels in *skl1-8* mutant compared with the wild type. We assume that the expression of nucleus-encoded but chloroplast-located proteins, is controlled by the anterograde and retrograde signals conveyed between the chloroplasts and the nucleus (Leister, 2005; Chi et al., 2013; Ng et al., 2014). Therefore, relative reduction in transcripts of nuclear-encoded genes in *skl1-8* is closely resembled to those of *embs* mutants, which can alter signaling level from chloroplast to nuclear and subsequently repress the expression of *RBCS* and *LHCBs* (Mochizuki et al., 2001; Rodermel, 2001; Strand et al., 2003; Chi et al., 2013). Previously, the two GLK transcription factors, AtGLK1 and AtGLK2 have been considered as positive regulators which can modulate the photosynthetic capacity by combining to the promoter, and the transcript levels of *GLK* genes can be affected by the state of plastid development (Kakizaki et al., 2009; Waters et al., 2009). In our results, we found that both *GLK1* and *GLK2* transcript levels were significantly reduced in the *skl1-8* mutant. This result coincided with previous study and suggested that *GLK* genes may perform as regulators in the expression of nuclear photosynthetic genes to cope with varying developmental and environmental conditions (Waters et al., 2009).

Most of induced genes can be classified as detoxification- and stress-related genes, including *peroxidase*, *WRKY* and *HSPs*. Presumably, the phenotype of *skl1-8* mutant is partially as result of side effects of photo-oxidative stress, and thus these genes may be induced for cell defense and survival under unfitted environmental conditions. Previous studies have indicated that HSPs and CPN play critical roles in the folding and protecting of enhanced *de novo* proteins from varied stresses (Zhang and Glaser, 2002). Interestingly, similar downregulated expression of photosynthetic genes had been reported under varied stress conditions (Bilgin et al., 2010), suggesting that the main defense response in plants is to depress photosynthesis capability in adverse environment to cope with the damages by oxidative molecular produced in plant cells.

In the *skl1-8* mutant, the chlorophyll and the carotenoid biosynthesis pathway related genes, such as *ATCLH2*, *PORs*, *BGL1*, *BETA-OHASE 2*, and *NCED4*, which also involved in the biosynthesis of secondary metabolites were downregulated. Similarly, the expression of genes involved in the related auxin pathways, such as *IAAs* and *PINs*, were significantly suppressed in *skl1-8* mutant. Previous research has revealed that auxin is a key regulator controlling bilateral symmetry and establishing embryo patterning in the process of embryogenesis (Liu et al., 1993). The altered expression of genes related to hormone signaling and secondary metabolites probably provided an explanation to the growth defects in *skl1-8* mutant.

## Author Contributions

JX designed the study. HX, LZ, RL, XW, SL, and XL performed the research. HX and JX analyzed the data and wrote the paper. YJ discussed the results and made comments on the manuscript.

## Conflict of Interest Statement

The authors declare that the research was conducted in the absence of any commercial or financial relationships that could be construed as a potential conflict of interest.

## Supplementary Material

TABLE S1 List of primers used in this study.

TABLE S2 Analysis of differentially expressed genes involved in the photosynthesis, pigment metabolism, carbon metabolism, biosynthesis of secondary metabolites, and plant hormone signal transduction.
