# Supplementary material for: SKL1 Is Essential for Chloroplast Development in Arabidopsis
Source: Front Plant Sci. 2018 Feb 20;9:179. doi: 10.3389/fpls.2018.00179 (PMC5826214; doi:10.3389/fpls.2018.00179)
Supplement: TABLE S1 — List of primers used in this study. [file Table_1.DOCX]

**Supplementary Table S1**

List of primers used in this study.

| **Gene ID** | **Primer name** | **Sequence (5′ to 3′)** | **Purpose** |
| --- | --- | --- | --- |
| AT3G27690 | *LHCB2.4*-qRT-F | GTGTGGTTCAAGGCAGGAT | RT |
|  | *LHCB2.4*-qRT-R | CGGGTAAAGCGGGTCAAG | RT |
| AT2G05100 | *LHCB2.1*-qRT-F | GAGAATACACCGTCCTACCTAAC | RT |
|  | *LHCB2.1*-qRT-R | GAGAACCTGCCTTGAACCA | RT |
| AT2G05070 | *LHCB2.2*-qRT-F | GTTCAAGGCAGGCTCTCA | RT |
|  | *LHCB2.2*-qRT-R | GTCCACCTCCGATTCTGTAG | RT |
| AT4G24280 | *cpHsc70-1*-qRT-F | CGAGGTTGTAGCGTTAGGT | RT |
|  | *cpHsc70-1*-qRT-R | AGTAGGCAGTGTGGTGTTC G | RT |
| AT3G09440 | *HSP70-3*-qRT-F | TACGGCTCAGACCACCAT | RT |
|  | *HSP70-3*-qRT-R | CACCAACAAGGACAACATCATC | RT |
| AT5G09590 | *MTHSC70-2*-qRT-F | CCTCTATCGCTCGGTATTGAA | RT |
|  | *MTHSC70-2*-qRT*-*R | GCTTGTTGTCTGTTGCCATT | RT |
| AT4G14690 | *ELIP2*-qRT-F | TGCCACAGTCTCCTCCTC | RT |
|  | *ELIP2*-qRT-R | CGCCACGAATCCAACCAT | RT |
| AT3G22840 | *ELIP1*-qRT-F | TCACCAGCACCATCTACCT | RT |
|  | *ELIP1*-qRT-R | CGCAACGAATCCAACCATC | RT |
| AT1G19670 | *ATCLH1*-qRT-F | ATCTTCGCAACTACTTCTACTCTG | RT |
|  | *ATCLH1*-qRT-R | GCATCGTCCACTTCCACTT | RT |
| AT4G19170 | *NCED4*-qRT-F | TCTTCTTCTTCCTTCCTCTCCT | RT |
|  | *NCED4*-qRT-R | GTGTGATTGGTTCGGTTGTG | RT |
| AT4G32280 | *IAA29*-qRT-F | TGGATGGTGTGGCAATAGC | RT |
|  | *IAA29*-qRT*-*R | GTATCTTCTCTGTCGCAATCTTCA | RT |
| AT3G15540 | *IAA19*-qRT-F | GCAGAGAAGATGATGAAGAAGAGA | RT |
|  | *IAA19*-qRT-R | CAGCGTCACCACCAGATG | RT |
| AT1G70940 | *PIN3*-qRT*-*F | TCTTCAACCACCACATCTACC | RT |
|  | *PIN3*-qRT*-*R | GAGTCGTCTGCTGATTCGTAT | RT |
| AT2G01420 | *PIN4*-qRT-F | CACTCTCCTTCTCTTCCTCTTC | RT |
|  | *PIN4*-qRT*-*R | CCTATCTCAGCATCCGTCTC | RT |
| AT4G14560 | *IAA1*-qRT-F | GGAGCACAAGAAGAACAACAAC | RT |
|  | *IAA1*-qRT*-*R | GCAGGAGGAGGAGCAGAT | RT |
| AT1G06950 | *ATTIC110*-qRT-F | AGTGGATGGTTATGATGCTGAA | RT |
|  | *ATTIC110*-qRT-R | CTGCTCTTGCTCGTCTGAT | RT |
| AT1G02280 | *TOC33*-qRT*-*F | ACCTTGCTTGTTCTGACTCAT | RT |
|  | *TOC33*-qRT-R | ATCTTCCGCTGTTCTCTGC | RT |
| AT3G54090 | *FLN1*-qRT-F | ACACTTCGGCTTCATTACTATACT | RT |
|  | *FLN1*-qRT-R | GTCGCTCCATCACATCCT | RT |
| AT1G69200 | *FLN2*-qRT-F | GATGTTCCTTGTCTGCTCCA | RT |
|  | *FLN2*-qRT*-*R | ACCTTCTTCTTCGTTGTTCTCTT | RT |

**RT-PCR., Reverse Transcription PCR;**
